# Efficient production of chimeric *Human papillomavirus *16 L1 protein bearing the M2e influenza epitope in *Nicotiana benthamiana *plants

**DOI:** 10.1186/1472-6750-11-106

**Published:** 2011-11-15

**Authors:** Slavica Matić, Riccardo Rinaldi, Vera Masenga, Emanuela Noris

**Affiliations:** 1 Istituto di Virologia Vegetale, CNR, Strada delle Cacce 73, 10135 Torino, Italy

## Abstract

**Background:**

*Human papillomavirus *16 (HPV-16) L1 protein has the capacity to self-assemble into capsomers or virus-like particles (VLPs) that are highly immunogenic, allowing their use in vaccine production. Successful expression of HPV-16 L1 protein has been reported in plants, and plant-produced VLPs have been shown to be immunogenic after administration to animals.

**Results:**

We investigated the potential of HPV-16 L1 to act as a carrier of two foreign epitopes from *Influenza A virus*: (i) M2e_2-24_, ectodomain of the M2 protein (M2e), that is highly conserved among all influenza A isolates, or (ii) M2e_2-9_, a shorter version of M2e containing the N-terminal highly conserved epitope, that is common for both M1 and M2 influenza proteins. A synthetic HPV-16 *L1 *gene optimized with human codon usage was used as a backbone gene to design four chimeric sequences containing either the M2e_2-24 _or the M2e_2-9 _epitope in two predicted surface-exposed L1 positions. All chimeric constructs were transiently expressed in plants using the *Cowpea mosaic virus*-derived expression vector, pEAQ-HT. Chimeras were recognized by a panel of linear and conformation-specific anti HPV-16 L1 MAbs, and two of them also reacted with the anti-influenza MAb. Electron microscopy showed that chimeric proteins made in plants spontaneously assembled in higher order structures, such as VLPs of T = 1 or T = 7 symmetry, or capsomers.

**Conclusions:**

In this study, we report for the first time the transient expression and the self-assembly of a chimeric HPV-16 L1 bearing the M2e influenza epitope in plants, representing also the first record of a successful expression of chimeric HPV-16 L1 carrying an epitope of a heterologous virus in plants. This study further confirms the usefulness of human papillomavirus particles as carriers of exogenous epitopes and their potential relevance for the production in plants of monovalent or multivalent vaccines.

## Background

Human papillomaviruses (HPVs) are non-enveloped tumour DNA viruses about 55 nm in diameter with a double-stranded circular genome of approximately 8 kb [1]. HPVs are the cause of approximately 5% of all cancers [2]. High risk HPVs, of which HPV-16 is the most prevalent, are responsible not only for causing cervical cancer in women [3] but also anogenital, head and neck tumours in men and women [4,5].

The major structural protein of HPV is the L1 capsid protein that can spontaneously self-assemble *in vivo *and *in vitro *to form capsomers and virus-like particles (VLPs) that are highly immunogenic [6,7]. L1 can form two types of VLPs, an icosahedral lattice with a T = 7 symmetry, composed of 72 pentamers which is morphologically identical to the native virions and a smaller T = 1 particle composed of 12 L1 pentamers [8,9]. The structure of the HPV16 T = 1 particle has been solved by crystallography. The L1 protein has a jellyroll β sandwich structure formed by conserved sequences with additional hypervariable loops protruding towards the outer surface of the capsid [9]. The three C-terminal helices 2, 3, and 4 (h2, h3, and h4) of L1 are surface exposed and their inter-capsomeric reactions influence VLP formation. The deletion of the full h4 has no impact on capsomer assembly, but prevents the formation of either T = 1 or T = 7 VLPs [10,11].

Recently prophylactic vaccines against HPV-16 have appeared on the market. These are composed of L1-based VLPs made in insect or yeast cells. Spontaneous assembly of HPV-16 L1 protein into VLPs has also been achieved in plants [12-14]. Plant-produced VLPs were immunogenic when administered to animals [15], further justifying continuing research into the use of plants for producing heterologous proteins for vaccine use. The HPV-16 L1 protein can assemble into VLPs in non-plant expression systems not only in its native form, but also in chimeric forms [16]; expressing the P1A peptide derived from the P815 tumour-associated antigen [17], an epitope of *Human immunodeficiency virus *type 1 [18], a short epitope of the hepatitis B core antigen [19], a neutralization epitope of the L2 minor capsid protein of HPV-6 and -16 [20], and a neutralizing epitope of *Human respiratory syncytial virus *(RSV) [21]. Chimeric VLPs have also been produced in transgenic and transplastomic plants [22-25].

Serological characterization of HPV-16 L1 particles has been carried out using monoclonal antibodies (MAbs) that recognize either linear or conformational surface-exposed epitopes. Amino acid (aa) residues 50, 266 and 282 of L1 are crucial for recognition by the conformation-specific and neutralizing MAbs H16:V5 and H16:E70 [26]. MAbs H16:7E, H16:9A, and H16:U4 recognize conformational epitopes which are not yet fully mapped (unidentified epitope within the aa region 1-173, for the first two MAbs, and aa 172-505 for the last MAb) [27]. Linear epitopes are recognized by MAbs H16:I23 (aa 111-130), H16:J4 (aa 261-280), and H16:D9 (unidentified epitope) [28]. These MAbs are fundamental to investigate the use of HPV-16 L1 as carrier, since they provide information of the intactness and the stability of L1-based structures.

For the purpose of expressing an HPV-16 L1-based scaffold in plants that can be easily engineered to carry foreign epitopes, we constructed a synthetic *L1ΔC22 *gene. To investigate the impact of the presence of a foreign epitope on VLP assembly and conformation, we designed four different synthetic genes encoding the L1 protein where two different L1 regions were substituted by either a 23-aa or a 8-aa epitope from the ectodomain of the M2 protein (M2e) of *Influenza A virus*. The chimeric L1 proteins generated were tested for their ability to form T = 1 or T = 7 particles, and characterized by their reactivity towards a panel of linear and conformation-specific anti HPV-16 L1 MAbs.

## Results

### Gene synthesis, cloning and protein structure modelling

**Figure 1:**
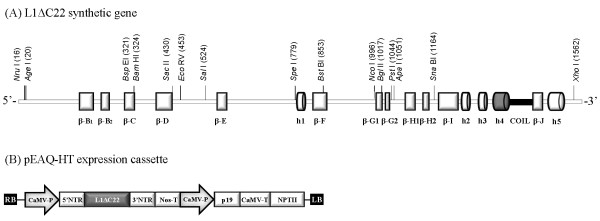
**Schematic representation of the synthetic *L1ΔC22 *gene and of the expressing cassettes made in the pEAQ-HT vector**. (a) Synthetic *L1ΔC22 *gene. Unique restriction sites with nucleotide positions in brackets are shown. Structural helices and β-sheets are indicated as cylinders or white boxes, respectively. The h4 or the coil region connecting h4 and β-J where the substitutions with the M2e epitopes were made are shown in gray or black, respectively. (b) pEAQ-HT expression cassette containing the *L1ΔC22 *gene, under the control of the *Cauliflower mosaic virus *35S promoter (CaMV-P), 5' and 3' non-translated regions (NTR) of *Cowpea mosaic virus *RNA-2, and nopaline synthase terminator (Nos-T). RB and LB: right and left borders of T-DNA, respectively; P19: suppressor of gene silencing; CaMV-T: CaMV terminator; NPT: neomycin phosphotransferase.

**Figure 2:**
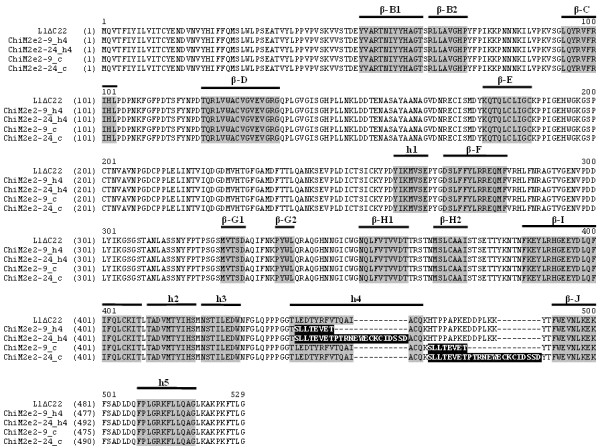
**Amino acid alignment of L1ΔC22 and chimeras**. Structural helices and β-sheets are indicated as shadowed areas. M2e_2-24 _(SLLTEVETPTRNEWECKCIDSSD) or M2e_2-9 _(SLLTEVET) epitopes are shown in black areas.

A synthetic *L1ΔC22 *gene lacking the 3'-terminal domain reported to code for a nuclear localization signal [29] was designed, that contained unique restriction sites located within nucleotide regions encoding the structural helices and β-sheets (Figure 1A). Restriction sites at the 5'- and 3'-terminus were introduced for appropriate cloning into the non-replicating plant expression vector pEAQ-HT (Figure 1B) [30]. Additionally, this gene sequence was optimized with human codon usage, as it was previously shown that this increased HPV-16 L1 yield in plants [12,14]. The *L1ΔC22 *gene sequence was used as a backbone to generate four chimeric variants (Table 1 and Figure 2) containing two different M2e influenza epitopes: M2e_2-24 _or M2e_2-9 _(aa 2-24 and aa 2-9 of the M2e protein, respectively) that replaced L1 sequences predicted to form either h4 (chimeras ChiM2e_2-24__h4 and ChiM2e_2-9__h4, respectively), or the coil region connecting h4 and β sheet J (hereinafter "coil") (chimeras ChiM2e_2-24__c and ChiM2e_2-9__c, respectively). *L1ΔC22 *and chimeric genes were cloned in pEAQ-HT, and after sequencing control, all constructs were transformed into *Agrobacterium tumefaciens*.

**Table 1:** Chimeric constructs with the L1ΔC22 regions deleted and the influenza epitopes inserted.

Chimeric variant	L1ΔC22 modifications				
	
	Structural function	Deleted sequence	aa position	Inserted Influenza M2e sequence	aa position
ChiM2e_2-24__h4	h4	LEDTYRFVTQAI	440-451	SLLTEVETPTRNEWECKCIDSSD	440-462
ChiM2e_2-9__h4	h4	LEDTYRFVTQAI	440-451	SLLTEVET	440-447
ChiM2e_2-24__c	coil between h4 and β-J	HTPPAPKEDDPLKK	456-469	SLLTEVETPTRNEWECKCIDSSD	456-478
ChiM2e_2-9__c	coil between h4 and β-J	HTPPAPKEDDPLKK	456-469	SLLTEVET	456-463

**Figure 3:**
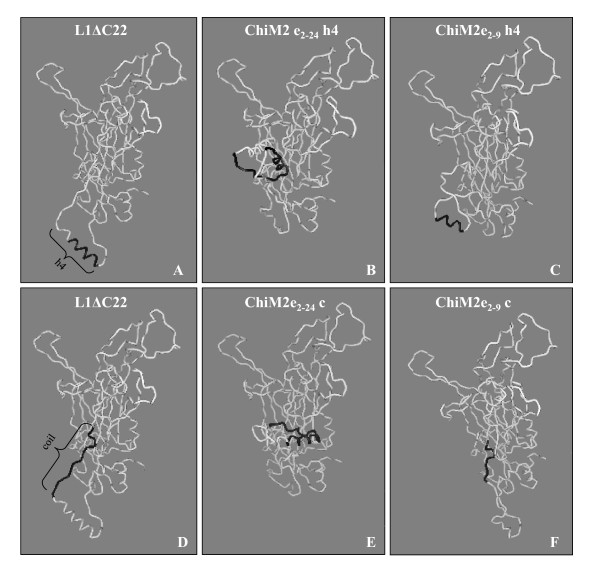
**Three-dimensional structure of L1ΔC22 (aa. 46-500) and chimeric monomers**. L1ΔC22 with marked (black) area within manipulated h4 or h4-β-J coil structure, where the substitution with the influenza M2e epitope was done (A and D); chimeras with their corresponding epitopes (in black) (B-C-E-F). Images A-B-C = structural modelling in h4; images D-E-F = structural modelling in the coil region. For monomer visualization, 3D-Mol Vector NTI suite 9 application was used.

Protein structure predictions revealed that all chimeras folded in a way that resembled the crystallographic structure of the native L1 protein [9], but we observed that chimeras with the shorter version of the M2e epitope replacing either h4 or the coil region were more similar than those carrying the longer M2e epitope version (Figure 3).

### Expression of L1ΔC22 and chimeric constructs

**Figure 4:**
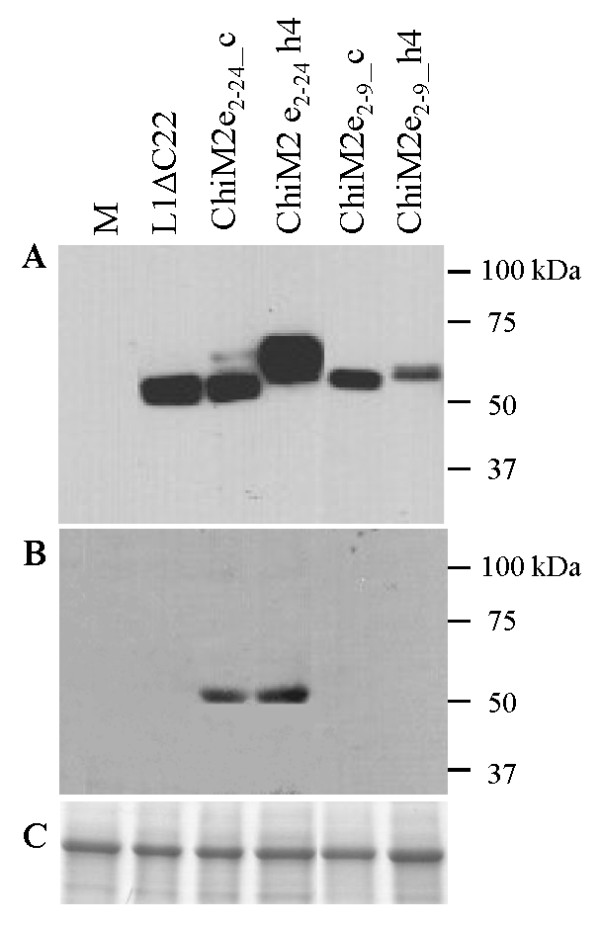
**Western blot analysis of leaf samples after infiltration with agrobacteria containing *L1ΔC22 *and the chimeric variants**. (A) Anti HPV16-L1 MAb MD2H11 was used at 1:5000 dilution. (B) Anti influenza Mab A M2 (14C2) was used at 1:1000 dilution. (C) Coomassie stained Rubisco large subunit served as a loading control. Molecular markers are indicated on the right. M is a leaf tissue extract infiltrated with buffer alone, used as a negative control.

To determine whether L1ΔC22 and its chimeric variants could be transiently expressed in *Nicotiana benthamiana*, we analysed tissue extracts from agroinfiltrated leaves by western blot using the MD2H11 MAb (kindly provided by M. Müller, German Cancer Research Center, Heidelberg, Germany) which is specific for the HPV16-L1 protein. *L1ΔC22 *and chimeric gene variants expressed an HPV-16 L1 monomer of the expected size of 55 kDa, with the exception of ChiM2e_2-24__h4 that migrated slightly slower and showed the strongest signal (Figure 4A). No evident proteolysis was observed with L1ΔC22 or with chimeric L1 protein extracts. Expression of L1ΔC22 and chimeras was confirmed with CAMVIR-1, another anti-HPV16 L1 MAb (data not shown). Testing for the presence of influenza epitopes using the anti-M2 14C2 MAb (Santa Cruz Biotechnology, Santa Cruz, CA) resulted in the recognition of the longer epitope (M2e_2-24_) in either h4 and the coil position, while this antibody was unable to detect constructs with the shorter epitope (M2e_2-9_) (Figure 4B). Thus we concluded that all synthetic chimeric constructs were successfully expressed and that two of them expressed also influenza epitopes. Further step was to determine if chimeras were able to preserve an appropriate conformation necessary to form assembled protein structures.

### Characterization of L1ΔC22 and chimeric constructs by capture-ELISA

**Figure 5:**
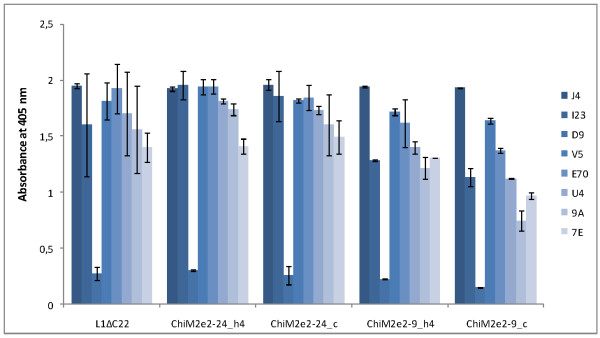
**Characterization of L1ΔC22 and chimeric variants expressed in *N. benthamiana *by capture-ELISA using linear and conformation-specific MAbs**. HPV-16 L1 VLPs derived from insect-cells were used as standard of known concentration (0.4 ng/μl). Absorbance values refer to 2 h after substrate addition.

All the linear and conformation-dependent MAbs reacted with L1ΔC22 and chimeric constructs from agroinfiltrated leaf extracts when analysed by capture ELISA (Figure 5). The highest L1 value was obtained six days post-agroinfiltration in a time course analysis of ten days (data not shown). Binding of conformation-specific MAbs with all L1-based chimeric protein constructs indicated that they aggregated in higher order structures, such as capsomers, or VLPs in either T = 1 or T = 7 form. Recognition of chimeras by these conformation-specific MAbs confirmed not only their expression in plants, but also proved that their self-assembly ability (into capsomer or VLP) was not abolished by the substitution of h4 or coil surface-exposed L1 regions with the foreign influenza epitopes. Among all chimeras, ChiM2e_2-24__h4 showed the highest binding affinity with the conformation-dependent neutralizing MAbs (H16:V5 and H16:E70).

The estimated yield of L1ΔC22 and chimeric proteins accumulated in agroinfiltrated leaves was measured six days post-agroinfiltration (Table 2). L1 quantification was calculated by capture-ELISA using the H16:J4 MAb which recognizes a linear epitope in both monomeric and assembled structures and the H16:V5 conformation-specific MAb which binds only to assembled structures. The highest L1 yield was obtained for ChiM2e_2-24__h4 with both MAbs (120 and 78 mg/kg plant material, with H16:J4 and H16:V5, respectively; corresponding to approximately 3.9% of total soluble proteins, TSP), followed by ChiM2e_2-24__c (45 mg/kg plant material, with both MAbs; corresponding to 1.5% TSP). ChiM2e_2-24__h4 accumulated also higher yield then the L1ΔC22 backbone protein, suggesting that the substituted amino-acid sequence had a positive impact on protein stability. On the other hand, the two chimeras bearing a shorter version of the M2e epitope accumulated at lower amount compared to both L1ΔC22 and chimeras having longer substitution. The greater difference observed between H16:J4 and H16:V5 in the case of L1ΔC22 and ChiM2e_2-24__h4 suggested that monomeric L1 protein was also accumulating, beside the assembled species.

**Table 2:** Concentration of L1ΔC22 and chimeric proteins

Antigen	Concentration in mg/kg calculated by capture-ELISA		TSP (%)
	
	H16:J4	H16:V5	
L1ΔC22	79	43	2.5
ChiM2e_2-24__h4	120	78	3.9
ChiM2e_2-9__h4	38	36	1.4
ChiM2e_2-24__c	45	45	1.5
ChiM2e_2-9__c	29	31	0.9

### Electron microscopy of purified L1ΔC22 and chimeric constructs

**Figure 6:**
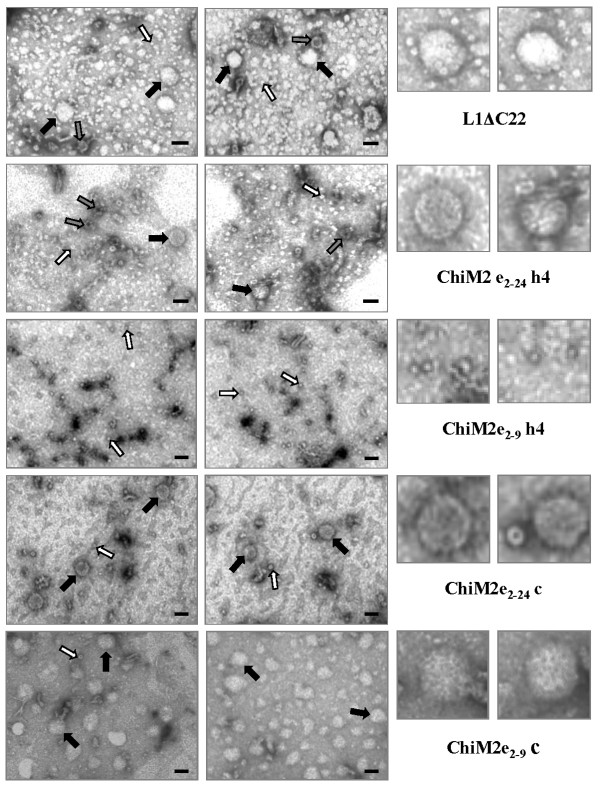
**Electron micrographs of purified bands from *N. benthamiana *leaf samples six days post-agroinfiltration with *L1ΔC22 *and chimeric constructs**. Black arrows = T7 VLPs of about 55 nm; grey arrows = T1 VLPs of about 30 nm; white arrows = capsomers. Assembled L1 structures for each constructs are enlarged on the right side. Bar = 50 nm.

To investigate the structure of VLPs and/or capsomers formed by L1ΔC22 and chimeras in plants, we carried out Cs_2_SO_4 _density gradient and further analysed the fractions obtained by electron microscopy. A begomovirus purification protocol [31] adopted for purifying VLPs of other papillomaviruses (Matić and Noris, unpublished results) was successfully employed to purify HPV-16 L1 particles. In L1ΔC22 preparations, spherical T = 7 VLPs of expected size of 55 nm were formed; in addition small VLPs with probable T = 1 symmetry of about 30 nm and capsomers ranging from 8-10 nm in diameter were also detected. Purified preparations of chimeric proteins showed that ChiM2e_2-24__c, ChiM2e_2-9__c, as well as ChiM2e_2-24__h4 successfully formed capsomers and VLPs of similar structure. On the contrary, the chimeric construct with the M2e_2-9 _substitution in h4 position (ChiM2e_2-9__h4) formed only capsomers (Figure 6).

Electron microscopy analysis confirmed the results of the capture-ELISA obtained with conformation-specific MAbs indicating the self-assembly of chimeric L1 protein in higher order protein structures (capsomers, T = 1 or T = 7 VLPs).

## Discussion

The current study supports the possibility of using plants as a method for the expression and folding of proteins of human viruses and raises the prospect of plants competing with other systems for the production of low-cost and effective vaccines. In this work, we demonstrated that: 1) the synthetic HPV-16 *L1 *gene can be successfully expressed in a transient manner in *N. benthamiana *plants using a non replicating pEAQ-HT expression vector; 2) the HPV-16 L1 carrier bearing the M2e influenza epitope can be efficiently produced in plants; 3) M2e influenza epitope presentation in either h4 or h4-β-J coil positions is not detrimental for the formation of capsomers and does not disrupt the recognition by conformation-dependent MAbs; 4) M2e epitopes in coil position allow VLP formation; 5) M2e epitopes in h4 position enable VLP assembly only when a longer sequence of amino acids is used.

The HPV-16 *L1 *gene and chimeras were expressed in plants using the small binary vector pEAQ-HT containing modified 5'- and 3'-untranslated regions from *Cowpea mosaic virus *(CPMV) RNA-2, avoiding the double cloning procedures necessary with standard binary vectors with a concomitant saving in time. The successful use of replicating viral vectors for the expression of heterologous proteins in plants has been reported on several occasions [32-34]. However, the size and complexity of viral vector systems, as well as the genetic stability of the constructs during viral genome replication and possible biocontainment issues can negatively affect their widespread use, justifying the selection of non-replicating pEAQ-HT plasmid as a vector of choice for the current studies.

The HPV-16 L1 protein carrier containing influenza epitopes was expressed in plants, but the recognition of M2e influenza sequences was achieved only in chimeras with the longer version of the epitope. It is yet to be investigated if the lack of reaction with constructs carrying the shorter version of the M2e influenza epitope is due to the use of an inappropriate MAb or to epitope inaccessibility on the carrier surface. So far, there is only one report of the expression of a chimeric HPV16-L1 protein in transgenic plants, where the chimera was made of L1 fused to E6/E7 epitopes of the same virus [22]. Here we achieved for the first time the transient expression in plants of chimeric HPV16-L1 bearing an epitope of a heterologous virus, with a substitution rather than a fusion strategy.

Several reports have demonstrated the usefulness of the M2e as a means of inducing neutralizing antibodies against influenza [35-37]. The N-terminus of M2e, containing the highly conserved SLLTEVET epitope, common to both M1 and M2 proteins, was sufficient to induce antibody production with an inhibitory effect against this pathogen [38]. Two different versions of this epitope were selected for the construction of HPV-chimeras. The major concern in introducing heterologous epitopes into the L1 platform is to ensure their surface-exposure while preserving the conformation of the L1 oligomer. HPV-16 pseudovirion infection is abolished by the neutralizing MAbs V5 and E70 which recognize surface exposed conformational epitopes of L1 [39]; these epitopes are considered of crucial importance for immunogenicity. All chimeric proteins tested in this work reacted positively with a panel of linear and conformation-specific MAbs. Furthermore, the positive reaction obtained with H16:V5 and H16:E70 indicated that both epitopes inserted in either L1 positions preserved these L1 immunogenic epitopes. Mab H16:D9 has affinity for the denatured VLP antigen [28], and the weak binding of L1ΔC22 and the chimeric proteins to this MAb demonstrates that the assembled products are stable.

Electron microscopy results showed that the L1 protein purified by Cs_2_SO_4 _density gradient centrifugation assembled into recognizable VLPs. Maclean et al. [14] transiently expressed the L1 protein in the cytoplasm and observed a size-range of the VLPs from 40-52 nm. Our data are consistent with this work, and we found VLPs ranging from 30-55 nm in the same plant host. Chimeric particles with M2e substitutions in the coil region predominantly assembled into T7 = VLPs. The substitution of 14 aa of the L1 coil region with either 8 or 23 aa of the Influenza M2e region was not detrimental for VLP formation, thus confirming that this region is not involved in the inter-pentameric reactions necessary to create a VLP. The chimeric polypeptides having shorter substitution in h4 (ChiM2e_2-9__h4) could still assemble into capsomers, but the formation of T = 1 or T = 7 VLPs was inhibited, as expected on the basis of the published crystal structure of HPV-16 L1 [10,11]. Interestingly, the substitution of the same h4 with a longer M2e sequence (ChiM2e_2-24__h4) allowed the assembly into T = 1 and T = 7 VLPs, along with capsomer formation, indicating that the incorporation of a longer version of the M2e epitope partially restored the function of the deleted h4 region. This suggests that the length of an epitope, as well as its amino acid sequence is important for efficient VLP formation. Furthermore, when h4 was substituted by a RSV epitope [21] or by a L2 epitope of HPV-6 and -16 [20], only capsomers were found assembled in preparations from insect cells systems. The detection of L1 superstructures for ChiM2e_2-24__h4 in plants suggests that the plant cell or the purification strategy could additionally play important role in HPV VLP formation/stability.

The yields reported in this work are in the range from 45 to 120 mg of total L1 protein per kg of fresh weight for ChiM2e_2-24__c and ChiM2e_2-24__h4 (on the basis of the linear H16:J4 MAb), thus fulfilling the often-requested quantity for commercially viable production of antibodies [15]. Previous works reported yields of L1 protein in *N. benthamiana *plants ranging from 40 μg per kg of plant material with a transient expression system of the native gene [40], to 533 mg per kg of plant material in the case of a chloroplast-targeted L1 protein transiently expressed from a human codon-optimized gene, with a 5-fold decrease when the NLS was removed (108 mg of L1ΔC22) [14]. Our yields of 79 mg for L1ΔC22 and 120 mg for ChiM2e_2-24__h4 are in accordance with the highest yields reported so far for plant-transiently expressed HPV-16 L1ΔC22. This approves the use of L1 gene with the human codon preferences and demonstrates the suitability of the pEAQ-HT vector for HPV-16 L1 transient expression in plants. Further enhancement of L1 yield could be obtained by appropriately decreasing the GC content, and by deleting regions prone to gene silencing, to reduce mRNA secondary structure.

Three of the four chimeric proteins tested assembled into VLPs and capsomers in plants, and one of them produced only capsomers. VLP assembly in the three chimeras emphasized their potential usefulness in future immunogenicity studies; in fact, to achieve comparable antibody responses at least 20 to 40 times more L1 are required in the form of capsomeres than in the form of VLPs [41]. Out of three VLP-forming chimeras, ChiM2e_2-24__h4 and ChiM2e_2-24__c reacted also with anti-influenza MAb, suggesting the availability of this epitope on VLP surface. Considering that these two chimeras had the highest L1 yield as well, we propose them as candidate multivalent vaccines for further immunological studies.

Our work demonstrated that chimeric HPV-16 L1 proteins can be transiently expressed in plants in only one week, with a significant-time reduction compared to other plant-expression systems (transgenic or transplastomic), thus offering new possibilities for rapid testing of new chimeras made in plants.

The use of a synthetic *L1 *gene designed to include unique restriction sites opens further perspectives for inserting other heterologous epitopes in different structural positions. Other interesting surface-exposed regions are the D-E and F-G1 loops for which the designed synthetic gene can be easily manipulated to present single or multiple foreign epitopes. This synthetic gene could also be used as a backbone for exchanging longer portions of other HPV-16 or related papillomavirus genes in order to synthesize novel vaccines with therapeutic and/or preventive use, or vaccines with multiple features effective against more than one papillomavirus.

## Conclusions

The HPV-16 L1 protein was successfully expressed by the CPMV-derived plant transient expression vector pEAQ-HT. Chimeric constructs bearing a heterologous influenza epitope were also successfully transiently expressed in plants as judged by their reaction with anti-HPV16 L1 specific MAbs. The characterization of chimeras by electron microscopy showed appropriate assembly of VLPs, with the exception of one chimera, which formed only capsomers. Chimeras were recognized by assembly-dependent conformation-specific MAbs, confirming particle stability. The substitution of the L1 h4 region with foreign epitopes of suitable size had a positive impact on L1 self-assembly into VLPs, compared to previous results showing that the deletion of the same h4 region abolished VLP assembly. Interestingly, this substitution had a positive effect on L1 yield, too. Moreover, we designed a synthetic gene allowing an easy manipulation of L1 structural positions suitable for inserting exogenous sequences, thus opening new perspectives for a rapid and easy construction of different-virus chimeras.

## Methods

### Gene synthesis and cloning

An HPV-16 *L1 *gene [GenBank:K02718] encoding the native protein sequence lacking 22 aa at the 3'-terminus was obtained by *de novo *synthesis from Geneart AG (Regensburg, Germany). The sequence encoding L1ΔC22 was optimized with human codon usage, and provided with restriction sites necessary for further cloning steps. The *L1ΔC22 *backbone gene was used to design four chimeric variants bearing two M2e influenza epitopes: M2e_2-24 _(SLLTEVETPTRNEWECKCIDSSD) or M2e_2-9 _(SLLTEVET) inserted in place of the L1 sequence in h4 or the coil region (Table 1).

*L1ΔC22 *and chimeric genes were excised from the pMK-RQ plasmid provided by GeneArt using the *Nru*I and *Xho*I restriction sites, and cloned in the corresponding sites of the CPMV-derived binary expression vector pEAQ-HT under the control of the *Cauliflower mosaic virus *35S promoter (kindly provided by G.P. Lomonossoff, John Innes Centre, Norwich, UK). Recombinant plasmids were transformed into *Escherichia coli *DH5α competent cells. The sequence of the resulting plasmids was verified by automatic sequencing at BMR Genomics (Padova, Italy). Multiple sequence alignment of nucleotide sequences (before and after synthesis) was done using AlignX (Vector NTI Suite V 5.5, InforMax, North Bethesda, Maryland) with the Clustal W algorithm [42].

### Protein structure generation and analysis

Based on the pentameric protein model obtained from HPV16-L1 T = 1 structure [PDB:1DZL], three-dimensional structure modelling of L1ΔC22 and chimeric variants was obtained using the 3D-JIGSAW Protein Comparative Modelling Server [43] [http://bmm.cancerresearchuk.org/~3djigsaw/]. 3D-Mol Vector NTI suite 9 application was used for protein visualization.

### Agroinfiltration of L1ΔC22 and chimeric constructs

*A. tumefaciens *LBA4404 was transformed with the plasmid pEAQ-HT containing *L1ΔC22 *and the four chimeras. Agrobacterium cultures were grown for 2 days at 28°C in YEB medium containing kanamycin (50 μg/ml) and rifampicin (50 μg/ml) and pelleted. After resuspension in 10 mM MES 2-(N-morpholino) ethansulfonic acid, pH 5.6, 10 mM MgCl_2_, and 100 μM acetosyringone to an OD_600 _of 0.8 and 3 h incubation at room temperature, bacterial suspensions were infiltrated into leaves of *N. benthamiana *plants. Control infiltration included the empty pEAQ-HT. Plants were maintained in a growth chamber at 23°C (16:8 h light:dark), and leaf tissue was collected six days after agroinfiltration.

### Protein extraction and analyses

Harvested agroinfiltrated tissue was ground in liquid N_2_, homogenized in 9 volumes of Laemmli sample buffer, and incubated at 100°C for 2 min. Extracts were separated on 7.5% mini-protean TGX gel (Bio-Rad, Richmond, CA) in Tris-glycine SDS-PAGE buffer, and either stained with Coomassie brilliant blue or electro-blotted to PVDF (polyvinylidene difluoride) membranes (Millipore, Billerica, MA) using transfer buffer (25 mM Tris, 192 mM glycine, 20% ethanol). The membranes were incubated overnight at 4°C in blocking solution (5% non-fat dry milk in PBS with 0.05% Tween-20), and incubated for 1 h at room temperature with primary antibody. After washing with PBS-0.05% Tween-20, the membranes were incubated for 1 h at room temperature with an anti-mouse horseradish peroxidase-conjugated secondary antibody (Sigma-Aldrich, St. Louis, MO) at a 1:10000 dilution. The reaction was detected with Supersignal West Pico Chemiluminescent Substrate (Thermo Fisher Scientific, Rockford, IL).

TSP were measured by a Bradford assay (Bio-Rad) following manufacturer's instructions.

### Characterization of L1ΔC22 and chimeric constructs by capture-ELISA

ELISA plates were coated overnight at 4°C with a 1:500 dilution of the anti-HPV-16 MAbs: H16:J4, H16:I23, H16:V5, H16:E70, H16:U4, H16:9A, H16:D9, H16:7E (kindly provided by N. Christensen, Pennsylvania State University College of Medicine, Hershey, PA), then washed and blocked with 5% milk PBS for 1 h at 37°C in. Extracts of soluble proteins were prepared by homogenizing the agroinfiltrated leaves in 10 volumes of high salt buffer (1× PBS, 0.5 M NaCl, 10 mM EDTA, 1 mM PMSF buffer). Extracts were incubated for 1 h at 37°C, followed by three washing steps. The 4543 polyclonal antibody obtained against HPV-16 VLPs (kindly provided by M. Müller) was added at a 1:3000 dilution in 5% milk PBS for 1 h at 37°C. Following three washings, the plates were incubated with an alkaline phosphatase-conjugated goat anti-rabbit antibody (1:10000, Sigma-Aldrich) for 1 h at 37°C. The reactions were detected using *p*-nitrophenylphosphate (Sigma-Aldrich), and absorbance of the reaction product measured at 405 nm with an ELISA microplate reader (Bio-Rad, model 3550). Insect cell-derived VLPs [41] of known concentrations were used as standards.

### Purification of L1ΔC22 and chimeric protein structures

VLPs and other higher order protein structures were purified from leaves agroinfiltrated with *L1ΔC22 *and chimeric constructs at six days post-agroinfiltration using a protocol for begomovirus purification, essentially as described by Luisoni et al. [31]. Harvested leaves were ground with liquid N_2_, homogenized in 5 vol of extraction buffer (0.5 M PB, pH 6.0 containing antioxidants, 2.5 mM EDTA, 1% Triton X-100, and 0.1% Driselase), and incubated overnight at 4°C. The homogenate was emulsified with 15% chloroform and centrifuged for 15 min at 8,000 × *g *(Sorvall SS-34 rotor; Du Pont, CT). The aqueous phase was collected and ultracentrifuged for 2 h at 205,000 × *g *(Beckman 55.2 Ti rotor; Beckman, CA). The resulting pellet was resuspended in 0.5 M PB (pH 7.0) containing 2.5 mM EDTA and centrifuged for 15 min at 8,000 × *g*. The supernatant was collected and loaded onto 20 to 50% Cs_2_SO_4 _density gradient in 0.5 M PB (pH 7.0) with 2.5 mM EDTA, and then subjected to ultracentrifugation for 5 h at 160,000 × *g *(Beckman SW41 rotor). Observed bands were collected, diluted in 0.1 M PB (pH 7.0), and ultracentrifuged for 40 min at 390,000 × *g *(Beckman TL100 rotor). Final pellets were resuspended in 0.1 M PB (pH 7.0) for electron microscopy observation.

### Electron microscopy of purified protein products

For electron microscopy analysis, the above obtained pelleted bands were adsorbed onto carbon-coated grid for approximately 1-3 minutes. Excess fluid was removed with filter paper and the grids were negatively stained with 0.5% uranyl acetate. The grids were then observed and photographed using a CM 10 electron microscope (Philips, The Netherlands).

## Authors' contributions

SM designed the synthetic genes, performed the bioinformatic analyses, cloning, the capture-ELISA, purification, and drafted the manuscript. RR carried out the agroinfiltration of plants, western blots and participated in purification assays. VM performed the electron microscopy work. EN conceived of the study, participated in its coordination and projection, and edited the manuscript. All authors have read and approved the final manuscript.
